# Genetic diversity and population structure of a rare flowering tree endemic to Appalachia, *Stewartia ovata*

**DOI:** 10.1002/ece3.11547

**Published:** 2024-06-25

**Authors:** L. K. Yadav, D. Bellis, Z. C. Smith, M. Ony, C. Hale, C. Richards, W. E. Klingeman, M. E. Staton, J. J. Granger, D. Hadziabdic

**Affiliations:** ^1^ Department of Entomology and Plant Pathology University of Tennessee Knoxville Tennessee USA; ^2^ Department of Plant Biology University of Georgia Athens Georgia USA; ^3^ Forest and Wildlife Research Center Mississippi State University Mississippi State Mississippi USA; ^4^ Department of Ecology and Evolution University of Chicago Chicago Illinois USA; ^5^ Department of Plant Sciences University of Tennessee Knoxville Tennessee USA

**Keywords:** conservation, genetic diversity, genotyping‐by‐sequencing, ornamental, *Stewartia ovata*

## Abstract

*Stewartia ovata* (cav.) Weatherby, commonly known as mountain stewartia, is an understory tree native to the southeastern United States (U.S.). This relatively rare species occurs in isolated populations in Virginia, Kentucky, Tennessee, North Carolina, South Carolina, Georgia, Alabama, and Mississippi. As a species, *S. ovata* has largely been overlooked, and limited information is available regarding its ecology, which presents obstacles to conservation efforts. *Stewartia ovata* has vibrant, large white flowers that bloom in summer with a variety of filament colors, suggesting potential horticultural traits prized by ornamental industry. However, *S. ovata* is relatively slow growing and, due to long seed dormancy, propagation is challenging with limited success rates. This has created a need to assess the present genetic diversity in *S. ovata* populations to inform potential conservation and restoration of the species. Here, we employ a genotyping‐by‐sequencing (GBS) approach to characterize the spatial distribution and genetic diversity of *S. ovata* in the southern Appalachia region of the eastern United States. A total of 4475 single nucleotide polymorphisms (SNPs) were identified across 147 individuals from 11 collection sites. Our results indicate low genetic diversity (He = 0.216), the presence of population structure (*K* = 2), limited differentiation (*F*_ST_ = 0.039), and high gene flow (Nm = 6.16) between our subpopulations. Principal component analysis corroborated the findings of STRUCTURE, confirming the presence of two distinct *S. ovata* subpopulations. One subpopulation mainly contains genotypes from the Cumberland Plateau, Tennessee, while the other consists of genotypes present in the Great Smoky Mountain ranges in Tennessee, North Carolina, and portions of Nantahala, Chattahoochee‐Oconee national forests in Georgia, highlighting that elevation likely plays a major role in its distribution. Our results further suggested low inbreeding coefficient (*F*_IS_ = 0.070), which is expected with an outcrossing tree species. This research further provides necessary insight into extant subpopulations and has generated valuable resources needed for conservation efforts of *S. ovata*.

## Graphical abstract

Stewartia ovata, commonly known as mountain stewartia, is a native shrub of the southeastern United States. Their growth is relatively slow and, due to long seed dormancy, they are difficult to propagate and their natural populations appears to be shrinking. Here, we employ a genotyping‐by‐sequencing (GBS) approach to characterize the genetic structure and diversity of mountain stewartia in the Smokey Mountain region of the eastern United States.

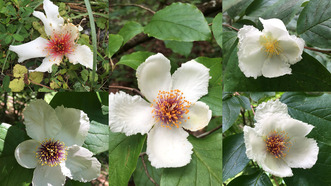

## 1 INTRODUCTION

**FIGURE 1:**
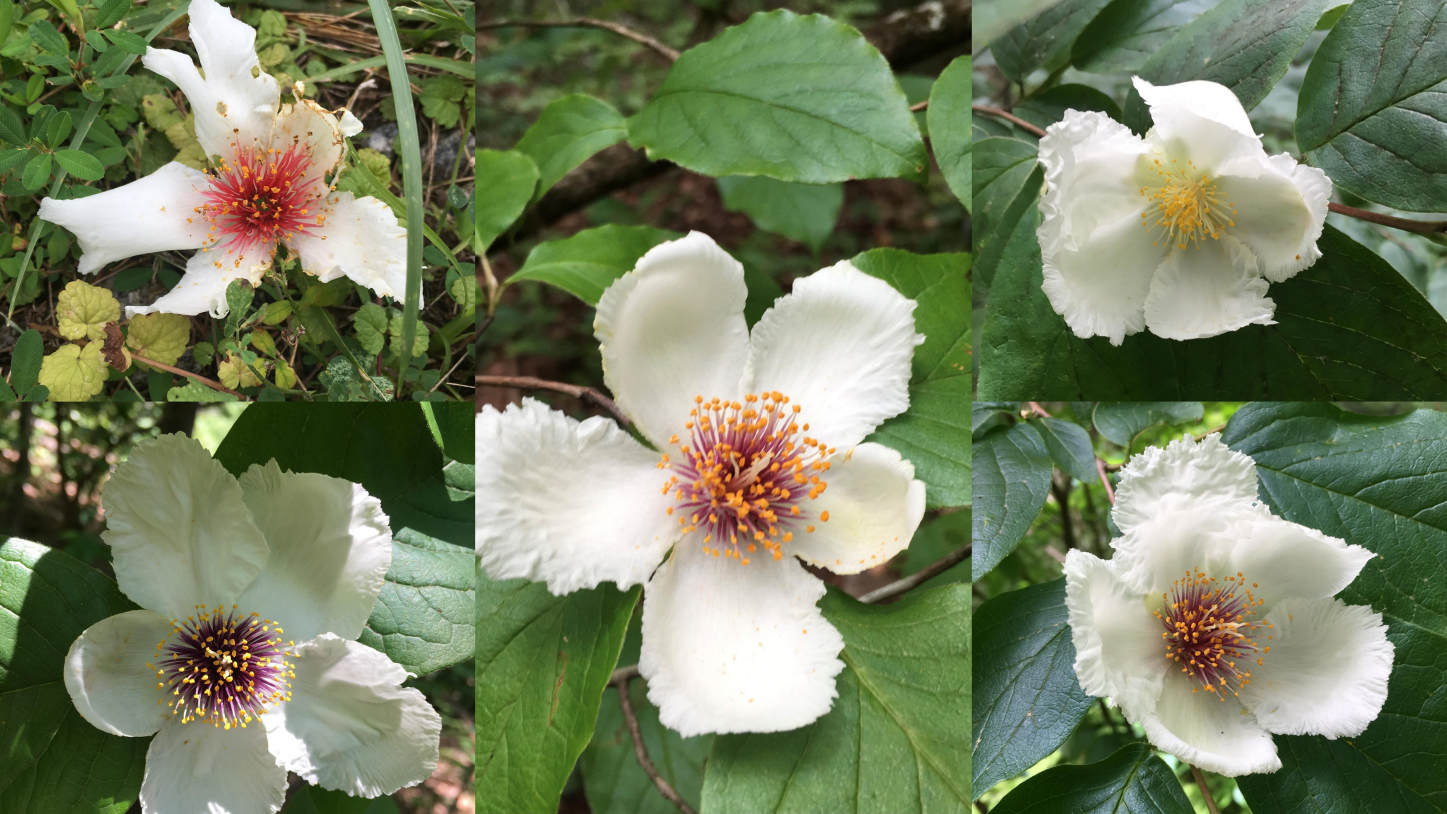
*Stewartia ovata* flower petals are showy and white while presenting a variety of stamen filament colors.

Endemic species have limited population size, and are often confined to a small geographical area, increasing the risk of peril. Endangered populations are usually more prone to inbreeding depression, fixation of deleterious alleles, and eventual extinction (Franklin, 1980; Simberloff, 1988). One such species present in the Great Smoky Mountain National Park (GSMNP) region (Tennessee and North Carolina), Cumberland Plateau area (Tennessee and Kentucky), and portions of Nantahala, Chattahoochee‐Oconee national forests (Georgia) is *Stewartia ovata* (cav.) Weatherby. The *Stewartia* genus is a member of the Theaceae, or Tea family, and comprises both evergreen and deciduous species distributed across the Asian to North American continents (Li et al., 2013; Prince, 2002). In the United States (U.S.), only two deciduous species, *S. ovata* (Cav.) Weatherby and *S. malacodendron*, are found, occurring in sparse populations across the southeastern United States (Spongberg & Fordham, 1975). *Stewartia ovata*, also known as mountain camelia, is a rare, deciduous, understory species indigenous to Kentucky, Tennessee, North and South Carolina, Georgia, Alabama, and Mississippi (Hale et al., 2022; Hobson & Houser, 2010; Spongberg, 1974) and grows from 300 to 800 m in elevation (Stupka, 1964; Swanson, 1994). There is also a small, isolated population of *S. ovata* at Chesapeake Bay National Estuarine Research Reserve in Virginia (Reay & Moore, 2009). *S. ovata* has many desirable, landscape esthetic properties like large white flowers (Figure 1) that possess a variety of stamen colors ranging from golden yellow to purple. The tree has a small architectural form (5–8 m), which can give specimens status as a prized landscape plant despite propagation challenges that constrain the availability of specimens in commercial production (Nair & Zhang, 2010). Sexual propagation of *S. ovata* is slow, with seeds typically taking 2–5 years to germinate. Asexual propagation is often unsuccessful due to the high mortality rate of rooted cuttings during overwintering (Curtis et al., 1996). In the wild, *S. ovata* primarily regenerates from decaying stumps of the same species (Granger et al., 2018).

Habitat loss and population fragmentation are the leading causes of ongoing biodiversity loss worldwide (Maxwell et al., 2016), which may cause genetic consequences like reduced ecological fitness of the species, poor seed/seedling health due to high inbreeding, and overall low genetic diversity (Frankham, 2005; Young et al., 1996). Beyond these factors, *S. ovata* has other biological constraints that may impact genetic diversity and ecological viability. Low overwinter survival, limited sexual propagation (Curtis et al., 1996), an overall increase in the incidence of pests and pathogens in southeastern forests (Lovett et al., 2016), and loss of habitat have raised concern for the long‐term survivability and persistence of *S. ovata* in the southeastern United States. This concern scenario mirrors a similar pattern observed with a close relative of *S. ovata* native to the southeastern United States, *Franklinia alatamaha* (Franklin tree) W. Bartram ex. Marshall (Gladfelter et al., 2020; Plummer, 1977). This species slowly became extinct from its native habitat in southeast Georgia, U.S., near the Altamaha River (Plummer, 1977). The cause of extinction is still unknown, but flooding and the invasive pathogen, *Phytophthora cinnamomi* Rands, are suspected to be driving factors (Gladfelter et al., 2020; Koslow & Peterson, 1980; Meyer et al., 2009; Prince, 1977). Due to the phylogenetic closeness of *S. ovata* and *F. alatamaha*, and its status as an ecologically narrow endemic, there is a cause for concern that *S. ovata* might suffer a similar fate. Hence, conservation efforts may be necessary to preserve *S. ovata* in the wild.

This study addresses the gap in knowledge surrounding the population genetic diversity of *S. ovata* and helps inform future conservation efforts. Low genetic diversity strongly restricts the ability of a species to adapt, expand, and overcome various environmental and ecological threats (Hendricks et al., 2017). Specifically, loss of genetic variation can result in inbreeding depression, reduced adaptability, and ultimately extinction of the species (Bruni et al., 2012). Species with isolated and highly fragmented populations also face higher levels of risk due to large levels of inbreeding and higher numbers of deleterious alleles (Charlesworth & Charlesworth, 1987). These factors will ultimately reduce the adaptive potential of the species and lower diversity further through genetic drift (Palsbøll et al., 2007). Assessment of adaptive and neutral genetic variation currently present in the population can also provide information about the local adaptation to inform regional and range‐wide conservation efforts (Flanagan et al., 2018). This information may directly impact future conservation decisions, such as assisted migration and genetic rescue.

An understanding of current geographical distribution, population structure, and genetic diversity can inform a level of conservation concern for a species and help to guide actions that are intended to enable preservation of maximum genetic diversity (Barbosa et al., 2018; Li et al., 2018). Currently, *S. ovata* is listed as “critically imperiled” in Mississippi; “imperiled” in Alabama, South Carolina, and Virginia; “vulnerable” in Georgia, North Carolina, and Kentucky; and “apparently secure” in Tennessee. This assessed conservation concern disparity apparent across the distributed range suggests that regional loss of biodiversity could be an important consideration in management strategies (NatureServe, 2023). Hence, a thorough examination of population genetic diversity of *S. ovata* in the southeastern United States will potentially help in long‐term preservation of the species.

Although genetic diversity studies of various Asian *Camelia* species closely related to *S. ovata* have been performed (Chang et al., 2017; Nair et al., 2005; Tang et al., 2006), our current understanding of *S. ovata* population diversity is limited. In general, genetic diversity studies have utilized microsatellites or amplified fragment length polymorphisms (AFLP) that offer fewer genetic loci for calculating diversity estimates, lower resolution, and limited transferability when compared with the genotyping‐by‐sequencing (GBS) approach used in this study. GBS is an effective approach for determining genetic variation on a genome‐wide scale, particularly in non‐model species that lack a sequenced genome (Peterson et al., 2012, 2014). To address this knowledge gap, our study objectives were the following: 1) to generate high‐density single nucleotide polymorphism (SNP) markers in wild populations of *S. ovata*, 2) to determine the level of extant genetic diversity across the distributed range for sampled specimens, and 3) to elucidate the patterns of admixture and across the spatial distribution of wild *S. ovata* populations.

For our study, we assessed the genetic diversity of 11 *S. ovata* subpopulations across the southeastern United States in the Cumberland Plateau region of Tennessee and the Great Smoky Mountain regions of North Carolina, Georgia, and Tennessee using GBS. The results generated here will not only serve to better inform conservation efforts of *S. ovata* but are also expected to inform future domestication efforts and help popularize this species as a prized native flowering deciduous tree species.

## 2 MATERIALS AND METHODS

### 2.1 Plant materials and sample collection

**FIGURE 2:**
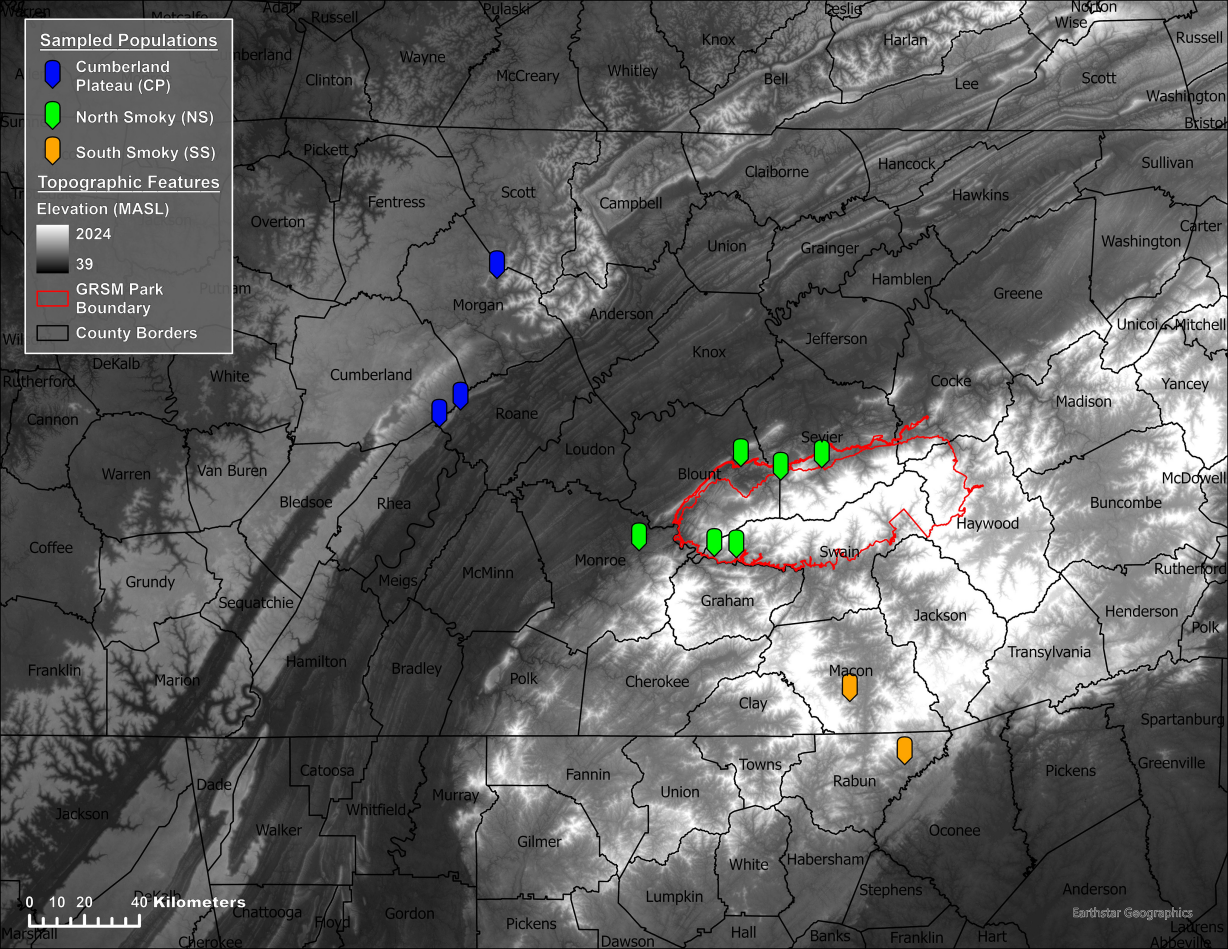
Map of collection sites of *Stewartia ovata*. Sampled tree specimens were located in three geographical regions: Cumberland Plateau (CP), North Smoky Mountains (NS), and South Smoky Mountains (SM).

*Stewartia ovata* samples were collected from 11 populations in the Cumberland Plateau, the Great Smoky Mountain National Park (GSMNP), portions of Nantahala, Chattahoochee‐Oconee national forests regions of Tennessee, North Carolina, and Georgia. Samples were collected from three geographical subdivisions at 11 collection sites, namely North Smoky Mountains (NS), 6 sites; South Smoky Mountains (SS), 2 sites; and Cumberland Plateau (CP), 3 sites (Figure 2; Table S1). The samples were comprised of 147 individual trees with 74 individuals collected from the northern region of the GSMNP, 21 from the southern region of the GSMNP, and 52 from the Cumberland Plateau (Table S1). The number of collection sites in each geographical subdivision was dictated by the availability and accessibility of *S. ovata* population in the region. Leaf tissues were collected from these trees and subsequently dried prior to DNA extraction.

### 2.2 DNA extraction and genotyping‐by‐sequencing

Approximately 100 mg of dried leaf tissue from each sample was used for DNA extraction. Tissue samples were homogenized with a Bead Mill 24 homogenizer (Fisher Scientific, Pittsburgh, Pennsylvania, USA), and DNA was extracted using the E.Z.N.A.® Plant DNA DS Mini Kit (Omega Bio‐Tek, Norcross, Georgia, USA) following the manufacturer's protocol with alterations described in Smith et al., 2023. Concentrations of gDNA were quantified using ND1000 Ultraviolet‐Vis Spectrophotometer (NanoDrop Technologies, Wilmington, Delaware, U.S.), and the gDNA was stored at −20°C until further use.

GBS was performed by the Biotechnology Center of the University of Wisconsin using the enzyme combination NsiI/BfaI. Illumina sequencing adaptors with unique barcode nucleotides were ligated to the digested DNA fragments to make libraries. The libraries were pooled together and initially sequenced on an Illumina MiSeq and then based on read ratios and rebalanced for equimolar concentration. The final balanced pool was sequenced on an Illumina NovaSeq 6000 (Illumina, San Diego, California, USA) generating 150 bp paired‐end reads for all samples. The raw sequence data have been deposited to NCBI under BioProject ID: PRJNA105204.

### 2.3 De novo sequence analysis

At this time, there is no publicly available *S. ovata* reference genome for aligning raw reads; hence, STACKS was used to process the reads. Process_radtags, ustacks, cstacks, sstacks, and populations programs in the STACKS software pipeline were used for de novo SNP discovery (Catchen et al., 2011). After data cleanup with Process_radtags, sequences were aligned to generate stacks with a minimum stack depth of 10 using the ustacks program. Next, cstacks were used to generate consensus loci for all genotypes, and allelic states of loci were determined by sstacks. Finally, SNP markers were generated from the catalog of alleles using the Populations program (Catchen et al., 2011). SNPs were then filtered with VCFtools (Danecek et al., 2011), using the following parameters: variants with a minimum quality score of 30, a minor allele count of at least 3, and a successful genotype in at least 50% of individuals. The SNPs were further filtered for a minimum read depth of five for downstream analysis.

### 2.4 Analysis of population structure

The population structure of *S. ovata* was determined using SNPs identified across 147 genotyped accessions. Three types of population structure analysis were performed: STRUCTURE (Pritchard et al., 2000), principal component analysis (PCA), and discriminant analysis of principal component (DAPC) (Jombart et al., 2010). DAPC differs from PCA as it overlooks within‐group variation to maximize between‐group variation. Genetic subpopulations of *S. ovata* were determined using STRUCTURE with the Bayesian admixture clustering model. STRUCTURE was run with a burn‐in period of 100,000 replications, a run length of 100,000 Markov Chain Monte Carlo (MCMC) iterations, and a number of subpopulations (K) iterations ranging from one to ten with 10 independent runs. The optimum K‐value was selected by calculating Delta K in Structure Harvester (Earl & VonHoldt, 2012) using the Evanno method. Next, PCA and DAPC analyses were conducted on SNP data using the POPPR 2.0 (Kamvar et al., 2014) and ADEGENET 2.1.7 (Jombart & Collins, 2015) packages in R version 4.1.3 (R Core Team, 2022).

### 2.5 Analysis of molecular variance and genetic diversity

The number of subpopulations determined by STRUCTURE and PCA was further used for analysis of molecular variance (AMOVA) calculations in POPPR 2.0 (Kamvar et al., 2014). Diversity analysis within *S. ovata* collection was evaluated using the R version 4.1.3. package snpR (Hemstrom & Jones, 2022) and ADEGENET 2.1.7 (Jombart & Collins, 2015), including the genetic fixation index (*F*_ST_), gene flow (Nm), inbreeding coefficient (*F*_IS_), pairwise *F*_ST_, expected heterozygosity (He), observed heterozygosity (Ho), and nucleotide diversity (pi) with default parameters.

## 3 RESULTS

### 3.1 SNP calling and filtering

GBS was performed on 147 *S. ovata* genotypes collected from the GSMNP, Cumberland Plateau, and portions of Nantahala, Chattahoochee‐Oconee national forests. A total of 885.9 million raw reads were obtained from 150 bp paired‐end sequencing. SNPs were detected and genotyped with STACKS de novo, yielding 11,908 SNP markers. After filtering, we retained 4475 high‐quality SNPs with 36% missing data for further downstream analyses. Among the filtered SNPs, we found more transitions (2495 loci, 55.75%) than transversions (1980 loci, 44.24%), yielding a ratio of transition/transversion of 1.26. The frequency of A/G (1245 loci, 27.82%) and C/T (1250 loci, 27.93%) transitions was similar. For the four transversions, we identified 867 A/C loci (19.37%), 480 A/T loci (10.72%), 343 G/T loci (7.66%), and 290 C/G loci (6.4%), respectively.

### 3.2 Population structure

**FIGURE 3:**
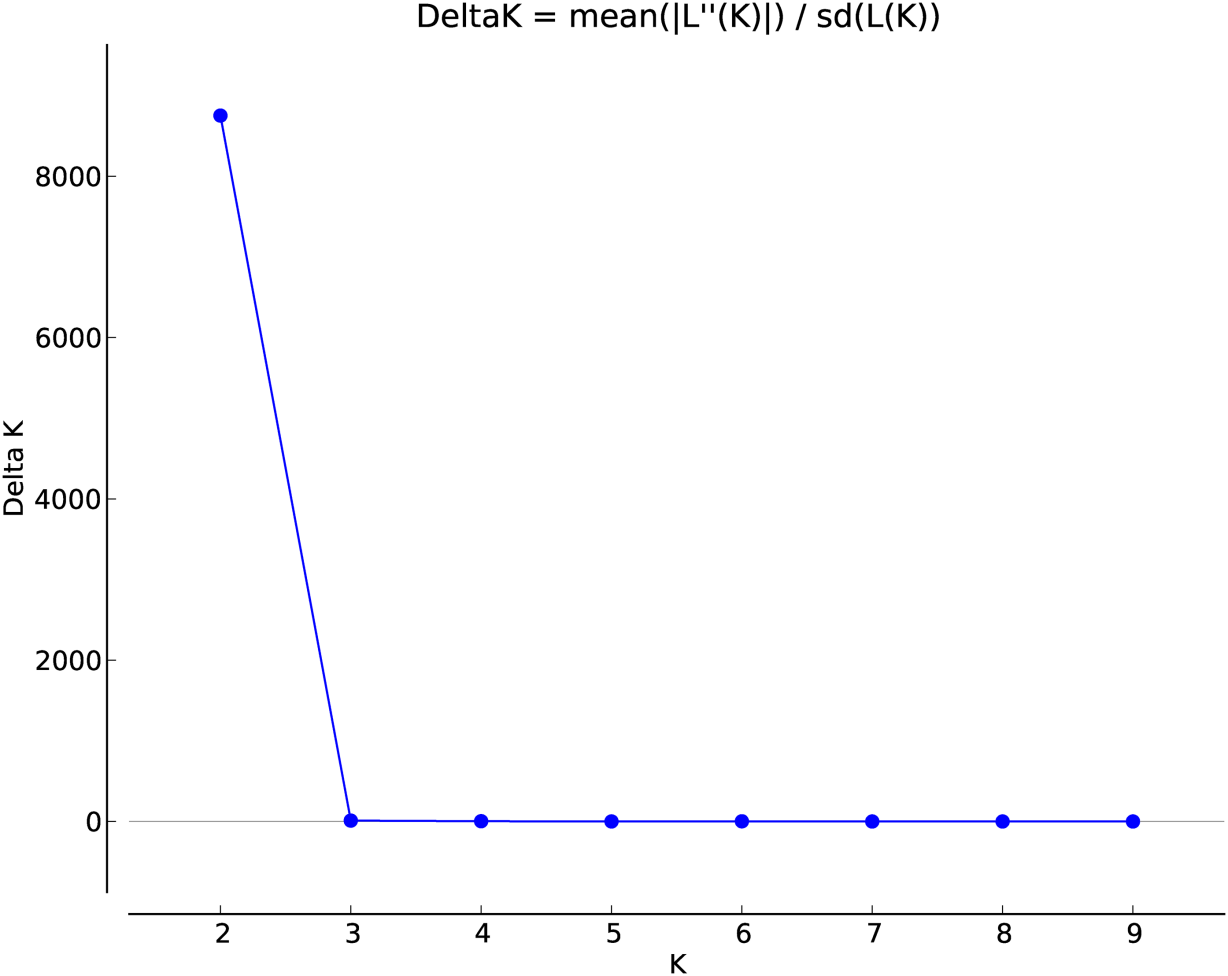
Delta K values for structure analysis across *Stewartia ovata* subpopulations.

**FIGURE 4:**
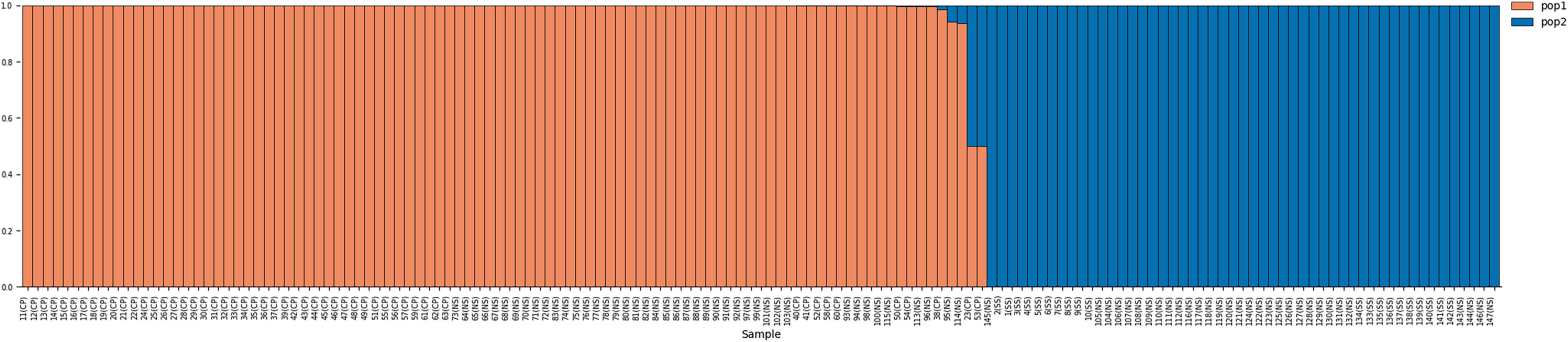
STRUCTURE bar graph of genetic clusters among three collection sites of *Stewartia ovata*. The color in the bar indicates the assignment probability of the sample to either of the two identified genetic clusters (orange = Pop1; blue = Pop2).

**FIGURE 5:**
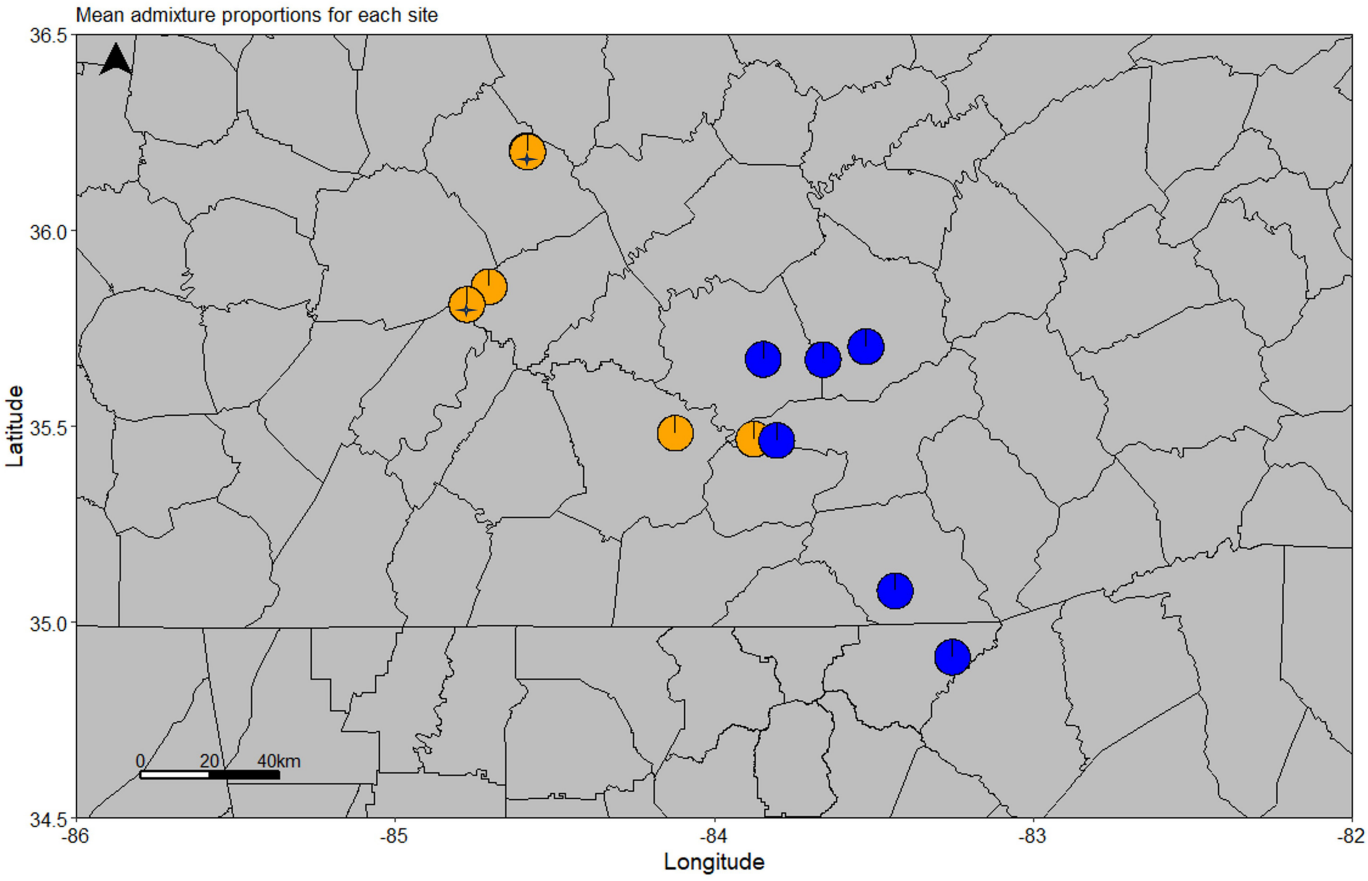
Admixture proportion across the collection sites on *Stewartia ovata* based on STRUCTURE results (orange = Pop1; blue = Pop2). The two admixtured genotypes are denoted by star (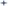) at their respective collection sites.

*Stewartia ovata* genotypes were collected from roughly three geographical regions including North Smoky Mountains (NS), South Smoky Mountains (SS), and Cumberland Plateau (CP). We used STRUCTURE, a model‐based method to analyze the genetic structure present in these geographical regions. STRUCTURE was run using 4475 SNPs and yielded two subpopulations, that is, biologically interpretable genetic clusters (Figure 3). Genotypes with admixture scores higher than 0.80 were assigned as pure members to their primary genetic cluster, while those with lower scores were considered admixed. Out of 147 genotypes, only two fell into the category of admixture, and the rest were separated into two clusters as pure members of a genetic cluster (Figure 4). In addition, data were further partitioned into three (delta *K* = 3), four (delta *K* = 4), and five (delta *K* = 5) genetic clusters, respectively, to examine divergence and admixture. However, additional cluster divisions did not provide clear geographical differentiation. Our results were best explained and supported by the presence of two subpopulation clusters in the surveyed area across three geographical regions (Figure 5). The subpopulations identified via STRUCTURE generally followed the geographical sampling. Samples from CP sites belong exclusively to Pop1, and samples from SS sites were exclusively assigned to Pop2. NS sites did not follow the expected geographical trend and included Pop1 and Pop2. However, individual NS sites tended to have all Pop1 or all Pop2 individuals. The two admixed individuals are located in the Cumberland Plateau (CP).

### 3.3 Analysis of molecular variance and genetic diversity

#### 3.3.1 Population genetic relationship

**FIGURE 6:**
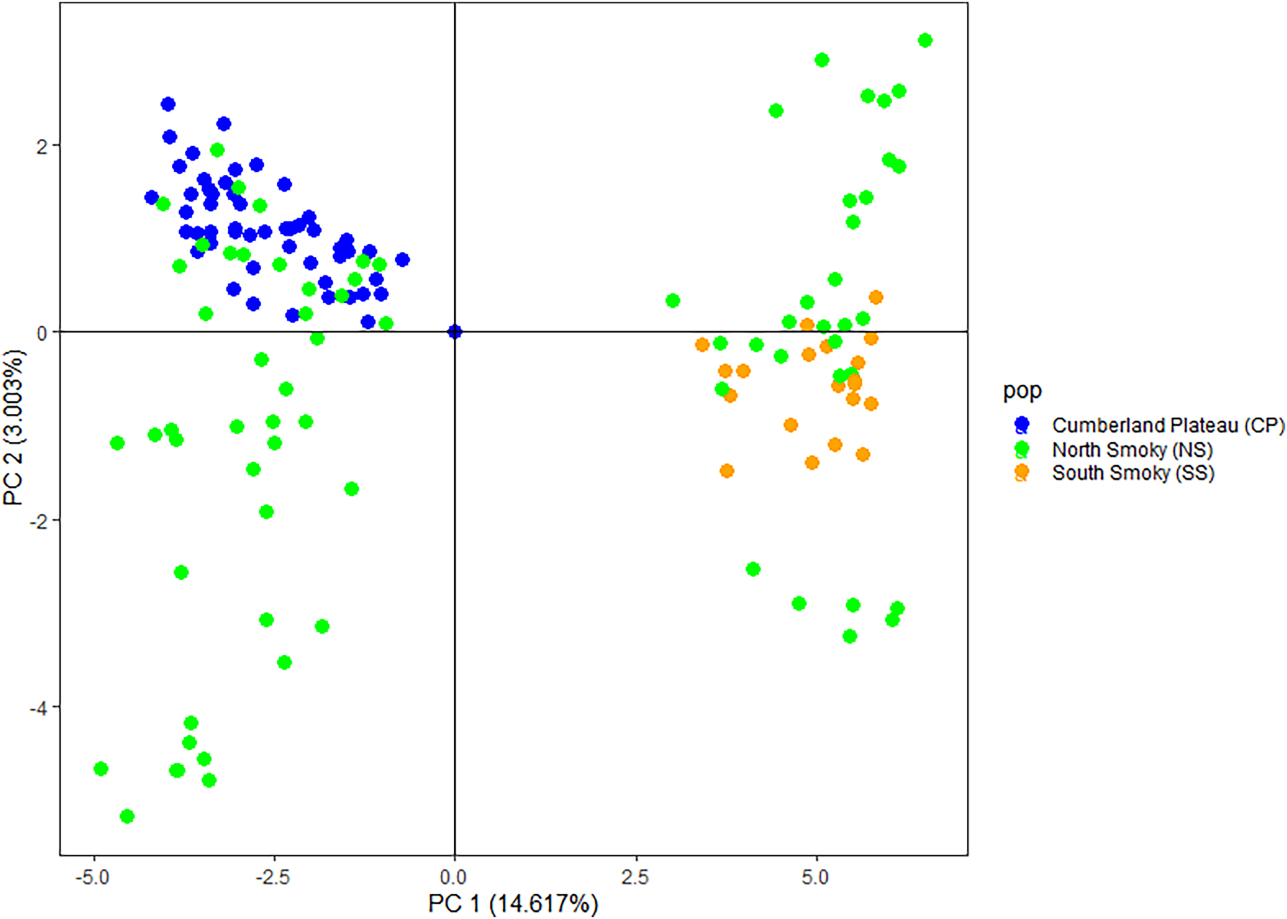
Principal component analysis (PCA) of 147 *Stewartia ovata* samples from three geographical collection sites based on 4475 SNPs that were derived from genotyping‐by‐sequencing analysis.

The same set of SNPs were analyzed using the non‐model‐based methods including PCA and DAPC. PCA and DAPC use multivariate approaches used to analyze genetic structure. While PCA analyzes both within‐ and between‐group variation, DAPC partitions out within‐group variation to maximize between‐group variation and describe genetic subpopulations. The PCA results echoed those of STRUCTURE and revealed the presence of two genetic clusters among *S. ovata* subpopulations (Figure 6). Four principal components were analyzed for PCA, and PC1 and PC2 explained more than 99% of the variance of the four components. The largest principal component, PC1, explained 14.61% of the variance, and it clearly separated genotypes from CP and SS with no bridging genotypes present in between these two subpopulations. The genotypes from NS also separated along PC1, clearly clustering with either CP or SS genotypes. In contrast, PC2 had a continuous distribution of genotypes and explained 3% of the variance. Interestingly, the NS genotypes had much wider variation along PC2 when compared to CP and SS.

**FIGURE 7:**
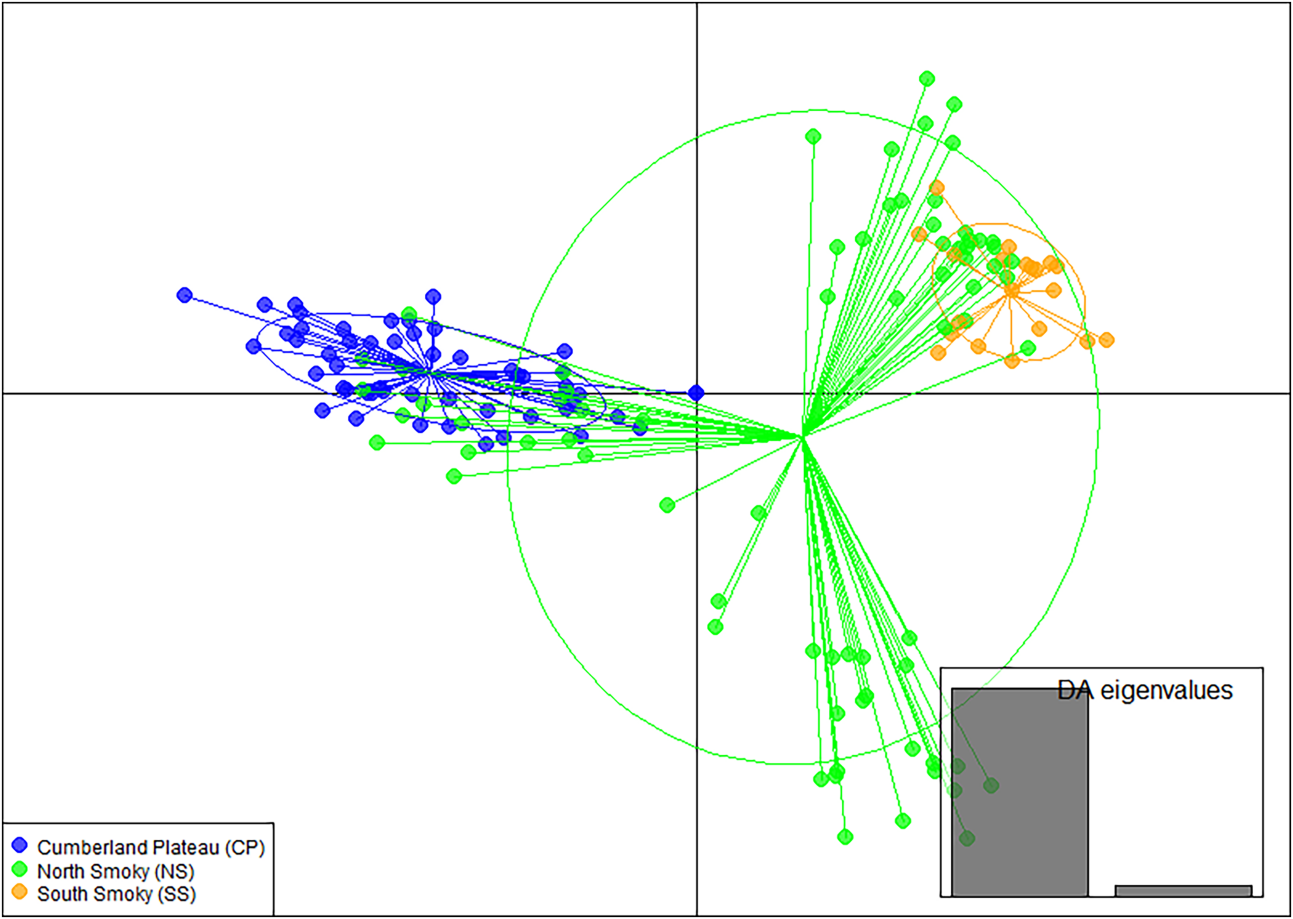
Discriminant analysis of principal components (DAPC) of *Stewartia ovata* population specimens. The scatter plot illustrates the first two linear discriminants identified by DAPC analysis. The figure depicts three collection sites. Circumferences surround each group, and small solid dots represent individual clones.

DAPC analysis (Figure 7) yielded results that were similar to PCA (Figure 6), yet DAPC analysis partitioned out, in‐group variation whereby the first two discriminant functions explained the majority of observed variance. The first DA (discriminant analysis eigenvalues on the X‐axis) of the DAPC identified the two population clusters of CP and SS previously identified by PCA and STRUCTURE, but with more bridging NS genotypes between the two clusters. Considering the first and second DAs together, a third cluster composed of a subset of NS genotypes emerges.

#### 3.3.2 Genetic diversity and population differentiation

The two subpopulations identified by STRUCTURE and PCA were used for AMOVA (Table 1). The analysis identified 11% variation between the two population clusters with high variation within samples (86%). The overall nucleotide diversity (pi) was 0.217 and nucleotide diversity for Pop1 (CP + NS) and Pop2 (SS + NS) was 0.204 and 0.203, respectively, indicating relatively constant diversity between the two population clusters. Our results indicated a low genetic differentiation (*F*_ST_ = 0.039), high gene flow (Nm = 6.16) between the populations, and a low inbreeding coefficient (F_IS_ = 0.07), suggesting predominantly out‐crossing population across *S. ovata* geographical distribution. Low inbreeding was also observed in the two individual subpopulations, with a *F*_IS_ for Pop1 (CP + NS) of 0.024 and for Pop2 (SS + NS) of −0.038 (Table 2).

**TABLE 1:** Analysis of molecular variance (AMOVA) of *Stewartia ovata* population members sampled.

Two clusters (Pop1 and Pop2)				
Source of variation	df	Sum of squares	Variation	Variation %
Between populations	1	9811.959	124.714	11.410
Between samples within populations	2	3186.349	18.120	1.657
Within samples	141	133,971.300	950.151	86.931
Total	144	146,969.607	1092.985	100.00

**TABLE 2:** Genetic diversity analysis of *Stewartia ovata* population STRUCTURE clusters.

Parameters	Overall	Pop1	Pop2
Nucleotide diversity	0.217	0.204	0.203
Expected heterozygosity	0.216	0.202	0.201
Observed heterozygosity	0.266	0.264	0.269
*F*_IS_	0.070	0.024	−0.038
Pairwise *F*_ST_	0.039		
Gene flow (Nm)	6.16		

## 4 DISCUSSION

Natural and anthropogenic habitat fragmentation disturbs and shapes modern landscapes around the world (LaBonte et al., 2017; Ramsfield et al., 2016). Plant populations present in these fragmented areas experience isolation and various abiotic and biotic stresses, such as poor reproductive success and reintroduction of deleterious alleles (Ewers & Didham, 2006; Fahrig, 2003). This fragmentation could cause reduced genetic diversity between subpopulations residing in these habitats resulting from an outcrossing mating system, limited gene flow, and reduced population size. Long‐term success of any population depends on its size and genetic diversity, and both factors are not in favor for the persistence of *S. ovata* in the wild. Our study of genetic diversity and population structure in *S. ovata* using GBS revealed a low genetic differentiation between the populations in the Smoky Mountain region and the Cumberland Plateau region of Tennessee. The population analysis highlighted the presence of two distinct but contiguous subpopulation clusters present in the region with extensive gene flow, which is consistent with other hardwood tree species in the region like flowering dogwood (*Cornus florida*) and viburnum (*Viburnum* spp.) (Dean et al., 2015; Hadziabdic et al., 2012). This differentiation coincides with the geographical differentiation of the region as elevation and human settlement plays a major role in these population clusters. The analysis further showed very little admixture between these two clusters. This is the first study of its kind in *S. ovata*, and our findings will serve as a key resource for the estimation of the present condition of genetic diversity in the wild population of this endemic and rare tree.

Effective management and conservation efforts of endangered species are highly dependent on the characterization of their genetic diversity and population structure. Aided by the introduction of affordable sequencing technologies like GBS, which generate densely packed genetic markers, researchers have been able to conduct population genomics studies on several types of forest trees such as pine (*Pinus* spp.), flowering dogwood (*C. florida*), and oak (*Quercus* spp.) (Li et al., 2023; Pais et al., 2020; Xiong et al., 2020). In the study reported here, we document the first population genetic analysis of *S. ovata* populations in the Cumberland Plateau and Great Smoky Mountain region of the southern United States achieved using a GBS approach (Narum et al., 2013). We identified 885.9 million reads and 4475 high‐quality SNPs, a result that is similar to recent studies in other tree species (Bentley et al., 2019; Korecký et al., 2021; Tsumura et al., 2020). The large number of SNP markers identified by GBS makes it a powerful tool for genetic diversity analysis (Niu et al., 2019; Pereira‐Dias et al., 2019). Among these SNPs, we found more transitions than transversions, as has been observed in previous studies in the tea family (Niu et al., 2019; Yang et al., 2016).

The outcome of our population structure analysis revealed the presence of two distinct subpopulations within *S. ovata*. When data were further partitioned into multiple genetic clusters (K values 3–5), our results were still best explained by the presence of two genetic clusters – higher K values did not indicate further population division or differentiation. While STRUCTURE identified two admixture genotypes, both in the Cumberland Plateau (CP) area, admixed individuals were not found using PCA. The third approach used in our analyses, DAPC, also loosely identified two population clusters with a subset of the NS group forming a plausible third group. This finding aligns with the topographic differences observed in the region. The genetic structure of any small and fragmented population is often shaped by local conditions like geography and human habitat (Zhu et al., 2016). The *S. ovata* population is scattered and fragmented due to the topographical variation (Hobson & Houser, 2010) and the presence of large human settlements between the Cumberland Plateau and the Smoky Mountain Region (Figure 2). These factors may have contributed to the genetic structure and low genetic diversity found in the region.

*F*_ST_, a measure of genetic differentiation (Frankham et al., 2002), revealed low genetic differentiation between the two subpopulations of *S. ovata* (*F*_ST_ = 0.04). This finding is consistent with other hardwood species including *C. florida* L. (*F*_ST_ = 0.07), *Cunninghamia lanceolata* (Lamb.) Hook. (*F*_ST_ = 0.04), and *Viburnum rufidulum* Raf. (*F*_ST_ = 0.06) (Dean et al., 2015; Duan et al., 2015; Hadziabdic et al., 2012; Hamrick & Godt, 1996; Hardesty et al., 2005). These findings were further supported by AMOVA where between‐population variation is only 11% and within‐sample variation is 86%. *S. ovata* genotypes in this study were from a small geographical region, which facilitates pollen and seed dispersal, resulting in detectable gene flow. The overall inbreeding coefficient is also low (0.070), indicating an outcrossing mode of reproduction and moderate connectivity between the two subpopulations. The inbreeding coefficient (*F*_IS_) of the subpopulations is also low (Pop1 (CP + NS) is 0.024 and Pop2 (SS + NS) is −0.038), further supporting the evidence for low genetic drift among the alleles in *S. ovata* subpopulations.

The nucleotide diversity and genetic diversity parameters also highlighted similar results between the two subpopulations. The overall nucleotide diversity was low (0.217), indicating a low mutation rate, population stability, and high gene flow within the *S. ovata* population in the region (Subramanian, 2019). The nucleotide diversity of two subpopulations was also low and similar to each other (Pop1 = 0.204; Pop2 = 0.203). The genetic diversity analysis also yielded similar results, and both Pop1 (0.264) and Pop2 (0.269) have similar levels of heterozygosity. Pop2 has slightly higher heterozygosity due to the more number and abundance of alleles compared to Pop1. The overall observed heterozygosity (H_O_ = 0.266) is higher than the expected heterozygosity (H_E_ = 0.216), indicating the presence of subpopulation clusters that may have been isolated in the past.

Isolated, small populations of species and individuals having low genetic diversity are less likely to adapt to changing environments and more likely to experience abiotic or biotic stressors leading to extinction due to selection pressure. *Stewartia ovata* has been observed to be environmentally temperamental, requiring specific ecological conditions like elevation and soil type for its growth and expansion apart from its poor reproduction and seed dispersal (Granger et al., 2018). This may lead to a reduction in population size which may be further compounded by human activity and loss of biodiversity due to global warming. Rapid biodiversity loss, physiological constraints, and evolutionary hazards have been major contributing factors for a species to become threatened or endangered (Hansen et al., 2001). *Stewartia ovata* is present in a protected national park, which may safeguard select subpopulations from habitat fragmentation, but this will not be sufficient to account for a changing climate. Global warming has caused the gradual loss of ideal habitat for many endemics (Nogué et al., 2009), and *S. ovata* is also not immune to this stressor. Furthermore, long‐term dormancy and poor seed germination rates may also contribute to a potential reduction of individuals in *S. ovata* populations.

According to our findings, there already is low genetic diversity present among wild growing *S. ovata* specimens and this condition may pose constraints on capacity of future breeding efforts. Necessary steps need to be taken now to preserve the present genetic diversity. One solution moving forward can be preserving this species by introducing individuals into a new location using plants representatives from both population clusters. Another mechanism that can sustain genetic diversity and access to the species is to popularize *S. ovata* as a potential native, small‐statured, flowering specimen tree or shrub for use in managed landscapes. *Stewartia ovata* has very desirable ornamental traits like showy flowers and a limited growth habit and architecture, as well as being native to the southeastern United States. Despite being a suitable candidate for landscaping and horticulture, it is relatively unknown by members of the general and gardening public due to constraints in reliable and efficient cultivation by experienced commercial growers. Therefore, we suggest breeding and conservation efforts to be directed toward preservation *S. ovata* in tandem with outreach to popularize this native tree species as a desirable native landscape specimen tree to extend the persistence and longevity of this species.

In conclusion, we accessed the genetic diversity of native understory shrub *S. ovata* subpopulations in the southeastern United States using GBS. This helped us identify two distinct subpopulations present in the region with low genetic diversity, limited differentiation, and extensive gene flow. Furthermore, *S. ovata* population has also been subjected to habitat loss. This may result in endangering the future of the species in the region and hinder future breeding efforts needed for species conservation.

## AUTHOR CONTRIBUTIONS

**L. K. Yadav:** Data curation (lead); formal analysis (lead); investigation (lead); methodology (equal); validation (lead); visualization (lead); writing – original draft (lead); writing – review and editing (equal). **D. Bellis:** Data curation (supporting); formal analysis (supporting); writing – review and editing (supporting). **Z. C. Smith:** Methodology (supporting); visualization (supporting); writing – review and editing (equal). **M. Ony:** Investigation (equal); methodology (lead); supervision (equal); writing – review and editing (equal). **C. Hale:** Methodology (equal); writing – review and editing (equal). **C. Richards:** Methodology (equal); writing – review and editing (equal). **W. E. Klingeman:** Conceptualization (lead); investigation (equal); methodology (equal); writing – review and editing (equal). **M. E. Staton:** Conceptualization (lead); funding acquisition (lead); investigation (equal); resources (equal); supervision (lead); writing – review and editing (equal). **J. J. Granger:** Conceptualization (lead); funding acquisition (lead); investigation (equal); methodology (equal); resources (equal); supervision (lead); writing – review and editing (equal). **D. Hadziabdic:** Conceptualization (lead); funding acquisition (lead); investigation (lead); methodology (lead); project administration (lead); resources (lead); supervision (lead); writing – review and editing (equal).

## CONFLICT OF INTEREST STATEMENT

None declared.

## Supporting information


Table S1.

## Data Availability

The data are available in NCBI under the BioProject no: PRJNA1052041.
