# Supplementary material for: Genetic diversity and population structure of a rare flowering tree endemic to Appalachia, Stewartia ovata
Source: Ecol Evol. 2024 Jun 25;14(6):e11547. doi: 10.1002/ece3.11547 (PMC11199121; doi:10.1002/ece3.11547)
Supplement: Supplementary file 1 — Table S1. [file ECE3-14-e11547-s001.docx]

**Supplementary Table 1:** Metadata for *Stewartia* collection sites.

| **Sample name** | **STRUCTURE Name** | **County** | **State** | **Site Name** | **Population Name** | **Latitude** | **Longitude** |
| --- | --- | --- | --- | --- | --- | --- | --- |
| 19_SO_1.1.1 | 11(CP) | Polk | Tennessee | Faust 1 | Cumberland Plateau (CP) | 35.19239722 | -84.58775833 |
| 19_SO_1.1.2 | 12(CP) | Polk | Tennessee | Faust 1 | Cumberland Plateau (CP) | 35.19239722 | -84.58775833 |
| 19_SO_1.1.3 | 13(CP) | Polk | Tennessee | Faust 1 | Cumberland Plateau (CP) | 35.19239722 | -84.58775833 |
| 19_SO_1.1.4 | 14(CP) | Polk | Tennessee | Faust 1 | Cumberland Plateau (CP) | 35.19239722 | -84.58775833 |
| 19_SO_1.1.5 | 15(CP) | Polk | Tennessee | Faust 1 | Cumberland Plateau (CP) | 35.19239722 | -84.58775833 |
| 19_SO_1.1.6 | 16(CP) | Polk | Tennessee | Faust 1 | Cumberland Plateau (CP) | 36.19222778 | -84.58780278 |
| 19_SO_1.2.1 | 17(CP) | Morgan | Tennessee | Faust 2 | Cumberland Plateau (CP) | 36.20380278 | -84.58780278 |
| 19_SO_1.2.2 | 18(CP) | Morgan | Tennessee | Faust 2 | Cumberland Plateau (CP) | 36.20391389 | -84.58782222 |
| 19_SO_1.2.3 | 19(CP) | Morgan | Tennessee | Faust 2 | Cumberland Plateau (CP) | 36.20391389 | -84.58782222 |
| 19_SO_1.2.4 | 20(CP) | Morgan | Tennessee | Faust 2 | Cumberland Plateau (CP) | 36.20391389 | -84.58782222 |
| 19_SO_1.2.5 | 21(CP) | Morgan | Tennessee | Faust 2 | Cumberland Plateau (CP) | 36.20391389 | -84.58782222 |
| 19_SO_1.2.6 | 22(CP) | Morgan | Tennessee | Faust 2 | Cumberland Plateau (CP) | 36.20391389 | -84.58782222 |
| 19_SO_1.3.1 | 30(CP) | Morgan | Tennessee | Faust 3 | Cumberland Plateau (CP) | 36.20035833 | -84.58594722 |
| 19_SO_1.3.2 | 31(CP) | Morgan | Tennessee | Faust 3 | Cumberland Plateau (CP) | 36.20044722 | -84.58571944 |
| 19_SO_1.3.3 | 32(CP) | Morgan | Tennessee | Faust 3 | Cumberland Plateau (CP) | 36.20044722 | -84.58571944 |
| 19_SO_1.3.5 | 33(CP) | Morgan | Tennessee | Faust 3 | Cumberland Plateau (CP) | 36.20054722 | -84.58578611 |
| 19_SO_1.3.6 | 34(CP) | Morgan | Tennessee | Faust 3 | Cumberland Plateau (CP) | 36.20054722 | -84.58578611 |
| 19_SO_1.3.7 | 35(CP) | Morgan | Tennessee | Faust 3 | Cumberland Plateau (CP) | 36.20053611 | -84.58568889 |
| 19_SO_1.3.8 | 36(CP) | Morgan | Tennessee | Faust 3 | Cumberland Plateau (CP) | 36.20052778 | -84.58578333 |
| 19_SO_1.3.10 | 23(CP) | Morgan | Tennessee | Faust 3 | Cumberland Plateau (CP) | 36.20040556 | -84.58581111 |
| 19_SO_1.3.11 | 24(CP) | Morgan | Tennessee | Faust 3 | Cumberland Plateau (CP) | 36.20040556 | -84.58581111 |
| 19_SO_1.3.12 | 25(CP) | Morgan | Tennessee | Faust 3 | Cumberland Plateau (CP) | 36.20038056 | -84.58585278 |
| 19_SO_1.3.13 | 26(CP) | Morgan | Tennessee | Faust 3 | Cumberland Plateau (CP) | 36.20038056 | -84.58585278 |
| 19_SO_1.3.14 | 27(CP) | Morgan | Tennessee | Faust 3 | Cumberland Plateau (CP) | 36.20034167 | -84.58585278 |
| 19_SO_1.3.15 | 28(CP) | Morgan | Tennessee | Faust 3 | Cumberland Plateau (CP) | 36.20034167 | -84.58585278 |
| 19_SO_1.3.16 | 29(CP) | Morgan | Tennessee | Faust 3 | Cumberland Plateau (CP) | 36.20034167 | -84.58585278 |
| 19_SO_2.1.1 | 37(CP) | Rhea | Tennessee | Rockwood 1 | South Smoky (SS) | 35.85625 | -84.70697222 |
| 19_SO_2.1.2 | 38(CP) | Rhea | Tennessee | Rockwood 1 | South Smoky (SS) | 35.85620833 | -84.70696944 |
| 19_SO_2.1.3 | 39(CP) | Rhea | Tennessee | Rockwood 1 | South Smoky (SS) | 35.85618611 | -84.70712778 |
| 19_SO_3.3.1 | 52(CP) | Rhea | Tennessee | Scarborough 3 | South Smoky (SS) | 35.81826111 | -84.78309722 |
| 19_SO_3.3.2 | 57(CP) | Rhea | Tennessee | Scarborough 3 | South Smoky (SS) | 35.81837778 | -84.78321111 |
| 19_SO_3.3.3 | 58(CP) | Rhea | Tennessee | Scarborough 3 | South Smoky (SS) | 35.81857778 | -84.78323056 |
| 19_SO_3.3.4 | 59(CP) | Rhea | Tennessee | Scarborough 3 | South Smoky (SS) | 35.81857778 | -84.78323056 |
| 19_SO_3.3.6 | 60(CP) | Rhea | Tennessee | Scarborough 3 | South Smoky (SS) | 35.81840278 | -84.7832 |
| 19_SO_3.3.7 | 61(CP) | Rhea | Tennessee | Scarborough 3 | South Smoky (SS) | 35.81840278 | -84.7832 |
| 19_SO_3.3.8 | 62(CP) | Rhea | Tennessee | Scarborough 3 | South Smoky (SS) | 35.81830278 | -84.78302778 |
| 19_SO_3.3.9 | 63(CP) | Rhea | Tennessee | Scarborough 3 | Cumberland Plateau (CP) | 35.81830278 | -84.78302778 |
| 19_SO_3.3.10 | 42(CP) | Roane | Tennessee | Scarborough 3 | Cumberland Plateau (CP) | 35.818075 | -84.78275833 |
| 19_SO_3.3.11 | 43(CP) | Rhea | Tennessee | Scarborough 3 | Cumberland Plateau (CP) | 35.81875833 | -84.78325556 |
| 19_SO_3.3.12 | 44(CP) | Rhea | Tennessee | Scarborough 3 | Cumberland Plateau (CP) | 35.81867778 | -84.78329722 |
| 19_SO_3.3.13 | 45(CP) | Rhea | Tennessee | Scarborough 3 | Cumberland Plateau (CP) | 35.81867778 | -84.78329722 |
| 19_SO_3.3.14 | 46(CP) | Rhea | Tennessee | Scarborough 3 | Cumberland Plateau (CP) | 35.81854444 | -84.78300278 |
| 19_SO_3.3.15 | 47(CP) | Rhea | Tennessee | Scarborough 3 | Cumberland Plateau (CP) | 35.81854444 | -84.78300278 |
| 19_SO_3.3.16 | 48(CP) | Rhea | Tennessee | Scarborough 3 | Cumberland Plateau (CP) | 35.81861944 | -84.78316389 |
| 19_SO_3.3.17 | 49(CP) | Rhea | Tennessee | Scarborough 3 | Cumberland Plateau (CP) | 35.81861944 | -84.78316389 |
| 19_SO_3.3.18 | 50(CP) | Rhea | Tennessee | Scarborough 3 | Cumberland Plateau (CP) | 35.81865556 | -84.78325278 |
| 19_SO_3.3.19 | 51(CP) | Rhea | Tennessee | Scarborough 3 | Cumberland Plateau (CP) | 35.81860278 | -84.78326389 |
| 19_SO_3.3.20 | 53(CP) | Rhea | Tennessee | Scarborough 3 | Cumberland Plateau (CP) | 35.81853056 | -84.78308056 |
| 19_SO_3.3.21 | 54(CP) | Rhea | Tennessee | Scarborough 3 | Cumberland Plateau (CP) | 35.81860833 | -84.78311111 |
| 19_SO_3.3.22 | 55(CP) | Rhea | Tennessee | Scarborough 3 | Cumberland Plateau (CP) | 35.81862778 | -84.78323333 |
| 19_SO_3.3.23 | 56(CP) | Rhea | Tennessee | Scarborough 3 | Cumberland Plateau (CP) | 35.81862778 | -84.78323333 |
| 19_SO_3.1.1 | 40(CP) | Roane | Tennessee | Scarborough 1 | Cumberland Plateau (CP) | 35.81086944 | -84.77623889 |
| 19_SO_3.1.2 | 41(CP) | Roane | Tennessee | Scarborough 1 | Cumberland Plateau (CP) | 35.81081667 | -84.77630833 |
| 19_SO_4.1.1 | 73(NS) | Monroe | Tennessee | Citico Creek 1 | Cumberland Plateau (CP) | 35.48244167 | -84.12315 |
| 19_SO_4.1.2 | 83(NS) | Monroe | Tennessee | Citico Creek 1 | Cumberland Plateau (CP) | 35.48244167 | -84.12315 |
| 19_SO_4.1.3 | 84(NS) | Monroe | Tennessee | Citico Creek 1 | Cumberland Plateau (CP) | 35.48237778 | -84.12325278 |
| 19_SO_4.1.5 | 85(NS) | Monroe | Tennessee | Citico Creek 1 | Cumberland Plateau (CP) | 35.48234722 | -84.12317222 |
| 19_SO_4.1.6 | 86(NS) | Monroe | Tennessee | Citico Creek 1 | Cumberland Plateau (CP) | 35.48234722 | -84.12317222 |
| 19_SO_4.1.7 | 87(NS) | Monroe | Tennessee | Citico Creek 1 | Cumberland Plateau (CP) | 35.48234722 | -84.12317222 |
| 19_SO_4.1.9 | 88(NS) | Monroe | Tennessee | Citico Creek 1 | Cumberland Plateau (CP) | 35.48215833 | -84.12313611 |
| 19_SO_4.1.10 | 64(NS) | Monroe | Tennessee | Citico Creek 1 | Cumberland Plateau (CP) | 35.48241944 | -84.12301667 |
| 19_SO_4.1.11 | 65(NS) | Monroe | Tennessee | Citico Creek 1 | Cumberland Plateau (CP) | 35.48258333 | -84.12301667 |
| 19_SO_4.1.12 | 66(NS) | Monroe | Tennessee | Citico Creek 1 | Cumberland Plateau (CP) | 35.48244444 | -84.12318611 |
| 19_SO_4.1.13 | 67(NS) | Monroe | Tennessee | Citico Creek 1 | North Smoky (NS) | 35.48245556 | -84.12296944 |
| 19_SO_4.1.14 | 68(NS) | Monroe | Tennessee | Citico Creek 1 | North Smoky (NS) | 35.48230556 | -84.12278889 |
| 19_SO_4.1.15 | 69(NS) | Monroe | Tennessee | Citico Creek 1 | North Smoky (NS) | 35.48230556 | -84.12278889 |
| 19_SO_4.1.16 | 70(NS) | Monroe | Tennessee | Citico Creek 1 | North Smoky (NS) | 35.48230556 | -84.12278889 |
| 19_SO_4.1.17 | 71(NS) | Monroe | Tennessee | Citico Creek 1 | North Smoky (NS) | 35.48233889 | -84.123 |
| 19_SO_4.1.18 | 72(NS) | Monroe | Tennessee | Citico Creek 1 | North Smoky (NS) | 35.48233889 | -84.123 |
| 19_SO_4.1.20 | 74(NS) | Monroe | Tennessee | Citico Creek 1 | North Smoky (NS) | 35.48242778 | -84.12291389 |
| 19_SO_4.1.21 | 75(NS) | Monroe | Tennessee | Citico Creek 1 | North Smoky (NS) | 35.48246944 | -84.12293333 |
| 19_SO_4.1.22 | 76(NS) | Monroe | Tennessee | Citico Creek 1 | North Smoky (NS) | 35.48239722 | -84.12313056 |
| 19_SO_4.1.23 | 77(NS) | Monroe | Tennessee | Citico Creek 1 | North Smoky (NS) | 35.48238889 | -84.12300278 |
| 19_SO_4.1.24 | 78(NS) | Monroe | Tennessee | Citico Creek 1 | North Smoky (NS) | 35.48258333 | -84.12257778 |
| 19_SO_4.1.25 | 79(NS) | Monroe | Tennessee | Citico Creek 1 | North Smoky (NS) | 35.48265833 | -84.12299722 |
| 19_SO_4.1.26 | 80(NS) | Monroe | Tennessee | Citico Creek 1 | North Smoky (NS) | 35.48238611 | -84.12288611 |
| 19_SO_4.1.27 | 81(NS) | Monroe | Tennessee | Citico Creek 1 | North Smoky (NS) | 35.48250556 | -84.12294444 |
| 19_SO_4.1.28 | 82(NS) | Monroe | Tennessee | Citico Creek 1 | North Smoky (NS) | 35.48239167 | -84.12271667 |
| 19_SO_5.1.1 | 93(NS) | Swain | North Carolina | 21 Mile Creek 1 | North Smoky (NS) | 35.46739167 | -83.87703333 |
| 19_SO_5.1.2 | 96(NS) | Swain | North Carolina | 21 Mile Creek 1 | North Smoky (NS) | 35.46739722 | -83.87703333 |
| 19_SO_5.1.3 | 97(NS) | Swain | North Carolina | 21 Mile Creek 1 | North Smoky (NS) | 35.46719444 | -83.87689444 |
| 19_SO_5.1.4 | 98(NS) | Swain | North Carolina | 21 Mile Creek 1 | North Smoky (NS) | 35.46711389 | -83.87675278 |
| 19_SO_5.1.5 | 99(NS) | Swain | North Carolina | 21 Mile Creek 1 | North Smoky (NS) | 35.46714722 | -83.87686389 |
| 19_SO_5.1.6 | 100(NS) | Swain | North Carolina | 21 Mile Creek 1 | North Smoky (NS) | 35.46735 | -83.87676111 |
| 19_SO_5.1.7 | 101(NS) | Swain | North Carolina | 21 Mile Creek 1 | North Smoky (NS) | 35.46739722 | -83.87674167 |
| 19_SO_5.1.8 | 102(NS) | Swain | North Carolina | 21 Mile Creek 1 | North Smoky (NS) | 35.46739722 | -83.87674167 |
| 19_SO_5.1.9 | 103(NS) | Swain | North Carolina | 21 Mile Creek 1 | North Smoky (NS) | 35.46736111 | -83.87691389 |
| 19_SO_5.1.10 | 89(NS) | Swain | North Carolina | 21 Mile Creek 1 | North Smoky (NS) | 35.46766944 | -83.87683611 |
| 19_SO_5.1.11 | 90(NS) | Swain | North Carolina | 21 Mile Creek 1 | North Smoky (NS) | 35.46798611 | -83.87663611 |
| 19_SO_5.1.13 | 91(NS) | Swain | North Carolina | 21 Mile Creek 1 | North Smoky (NS) | 35.46798611 | -83.87663611 |
| 19_SO_5.1.16 | 92(NS) | Swain | North Carolina | 21 Mile Creek 1 | North Smoky (NS) | 35.46798611 | -83.87663611 |
| 19_SO_5.1.21 | 94(NS) | Swain | North Carolina | 21 Mile Creek 1 | North Smoky (NS) | 35.46922778 | -83.87569444 |
| 19_SO_5.1.22 | 95(NS) | Swain | North Carolina | 21 Mile Creek 1 | North Smoky (NS) | 35.46922778 | -83.87569444 |
| 19_SO_7.1.1 | 113(NS) | Sevier | Tennessee | West Prong 1 | North Smoky (NS) | 35.70325833 | -83.52559167 |
| 19_SO_7.1.2 | 114(NS) | Sevier | Tennessee | West Prong 1 | North Smoky (NS) | 35.70295833 | -83.52577778 |
| 19_SO_7.1.3 | 115(NS) | Sevier | Tennessee | West Prong 1 | North Smoky (NS) | 35.70319444 | -83.52551389 |
| 19_SO_7.1.4 | 116(NS) | Sevier | Tennessee | West Prong 1 | North Smoky (NS) | 35.70331667 | -83.52570556 |
| 19_SO_7.1.5 | 117(NS) | Sevier | Tennessee | West Prong 1 | North Smoky (NS) | 35.70325556 | -83.52576111 |
| 19_SO_7.1.6 | 118(NS) | Sevier | Tennessee | West Prong 1 | North Smoky (NS) | 35.70340833 | -83.52578056 |
| 19_SO_7.1.7 | 119(NS) | Sevier | Tennessee | West Prong 1 | North Smoky (NS) | 35.70355278 | -83.52556667 |
| 19_SO_7.1.8 | 120(NS) | Sevier | Tennessee | West Prong 1 | North Smoky (NS) | 35.70351389 | -83.52562778 |
| 19_SO_7.1.9 | 121(NS) | Sevier | Tennessee | West Prong 1 | North Smoky (NS) | 35.70373611 | -83.52525 |
| 19_SO_8.1.1 | 124(NS) | Sevier | Tennessee | Sinks 1 | North Smoky (NS) | 35.67023889 | -83.65993056 |
| 19_SO_8.1.2 | 125(NS) | Sevier | Tennessee | Sinks 1 | North Smoky (NS) | 35.67023889 | -83.65993056 |
| 19_SO_8.1.3 | 126(NS) | Sevier | Tennessee | Sinks 1 | North Smoky (NS) | 35.67032778 | -83.66008889 |
| 19_SO_8.1.4 | 127(NS) | Sevier | Tennessee | Sinks 1 | North Smoky (NS) | 35.67032778 | -83.66008889 |
| 19_SO_8.1.5 | 128(NS) | Sevier | Tennessee | Sinks 1 | North Smoky (NS) | 35.67032778 | -83.66008889 |
| 19_SO_8.1.6 | 129(NS) | Sevier | Tennessee | Sinks 1 | North Smoky (NS) | 35.67017222 | -83.65995278 |
| 19_SO_8.1.7 | 130(NS) | Sevier | Tennessee | Sinks 1 | North Smoky (NS) | 35.67017222 | -83.65995278 |
| 19_SO_8.1.8 | 131(NS) | Sevier | Tennessee | Sinks 1 | North Smoky (NS) | 35.67014444 | -83.66029444 |
| 19_SO_8.1.9 | 132(NS) | Sevier | Tennessee | Sinks 1 | North Smoky (NS) | 35.67014444 | -83.66029444 |
| 19_SO_8.1.10 | 122(NS) | Sevier | Tennessee | Sinks 1 | North Smoky (NS) | 35.67043889 | -83.66014167 |
| 19_SO_8.1.11 | 123(NS) | Sevier | Tennessee | Sinks 1 | North Smoky (NS) | 35.67042222 | -83.66038611 |
| 19_SO_6.2.1 | 105(NS) | Swain | North Carolina | Fontana Dam 1 | North Smoky (NS) | 35.46375 | -83.80502778 |
| 19_SO_6.2.2 | 106(NS) | Swain | North Carolina | Fontana Dam 1 | North Smoky (NS) | 35.46375 | -83.80502778 |
| 19_SO_6.2.3 | 107(NS) | Swain | North Carolina | Fontana Dam 1 | North Smoky (NS) | 35.463925 | -83.80519444 |
| 19_SO_6.2.4 | 108(NS) | Swain | North Carolina | Fontana Dam 1 | North Smoky (NS) | 35.46379444 | -83.80481111 |
| 19_SO_6.2.5 | 109(NS) | Swain | North Carolina | Fontana Dam 1 | North Smoky (NS) | 35.46366944 | -83.80488889 |
| 19_SO_6.2.6 | 110(NS) | Swain | North Carolina | Fontana Dam 1 | North Smoky (NS) | 35.46384167 | -83.80472778 |
| 19_SO_6.2.7 | 111(NS) | Swain | North Carolina | Fontana Dam 1 | North Smoky (NS) | 35.45408056 | -83.80489167 |
| 19_SO_6.2.9 | 112(NS) | Swain | North Carolina | Fontana Dam 1 | North Smoky (NS) | 35.46384722 | -83.80475833 |
| 19_SO_6.2.10 | 104(NS) | Swain | North Carolina | Fontana Dam 1 | North Smoky (NS) | 35.46375556 | -83.80469722 |
| 19_SO_9.1.1 | 134(SS) | Macon | North Carolina | North Fork Creek 1 | North Smoky (NS) | 35.07994167 | -83.43398611 |
| 19_SO_9.1.2 | 135(SS) | Macon | North Carolina | North Fork Creek 1 | North Smoky (NS) | 35.07994167 | -83.43398611 |
| 19_SO_9.1.3 | 136(SS) | Macon | North Carolina | North Fork Creek 1 | North Smoky (NS) | 35.07994167 | -83.43398611 |
| 19_SO_9.1.4 | 137(SS) | Macon | North Carolina | North Fork Creek 1 | North Smoky (NS) | 35.07994167 | -83.43398611 |
| 19_SO_9.1.5 | 138(SS) | Macon | North Carolina | North Fork Creek 2 | North Smoky (NS) | 35.07994444 | -83.43398611 |
| 19_SO_9.1.6 | 139(SS) | Macon | North Carolina | North Fork Creek 3 | North Smoky (NS) | 35.07994722 | -83.43398611 |
| 19_SO_9.1.7 | 140(SS) | Macon | North Carolina | North Fork Creek 1 | North Smoky (NS) | 35.07994167 | -83.43398611 |
| 19_SO_9.1.8 | 141(SS) | Macon | North Carolina | North Fork Creek 1 | North Smoky (NS) | 35.07994167 | -83.43398611 |
| 19_SO_9.1.9 | 142(SS) | Macon | North Carolina | North Fork Creek 1 | North Smoky (NS) | 35.07994167 | -83.43398611 |
| 19_SO_9.1.10 | 133(SS) | Macon | North Carolina | North Fork Creek 1 | North Smoky (NS) | 35.07994167 | -83.43398611 |
| 19_SO_10.1.1 | 2(SS) | Rabun | Georgia | Sarah's Creek 1 | South Smoky (SS) | 34.91084444 | -83.25448889 |
| 19_SO_10.1.2 | 3(SS) | Rabun | Georgia | Sarah's Creek 2 | South Smoky (SS) | 34.91144167 | -83.2541 |
| 19_SO_10.1.3 | 4(SS) | Rabun | Georgia | Sarah's Creek 3 | South Smoky (SS) | 34.91140556 | -83.25393333 |
| 19_SO_10.1.4 | 5(SS) | Rabun | Georgia | Sarah's Creek 4 | South Smoky (SS) | 34.91146111 | -83.25357778 |
| 19_SO_10.1.5 | 6(SS) | Rabun | Georgia | Sarah's Creek 5 | South Smoky (SS) | 34.911575 | -83.253575 |
| 19_SO_10.1.6 | 7(SS) | Rabun | Georgia | Sarah's Creek 6 | South Smoky (SS) | 34.91140556 | -83.25349167 |
| 19_SO_10.1.7 | 8(SS) | Rabun | Georgia | Sarah's Creek 7 | South Smoky (SS) | 34.91135556 | -83.25339167 |
| 19_SO_10.1.8 | 9(SS) | Rabun | Georgia | Sarah's Creek 8 | South Smoky (SS) | 34.91126389 | -83.25419444 |
| 19_SO_10.1.9 | 10(SS) | Rabun | Georgia | Sarah's Creek 9 | South Smoky (SS) | 34.90872778 | -83.25378889 |
| 19_SO_10.1.10 | 1(SS) | Rabun | Georgia | Sarah's Creek 10 | South Smoky (SS) | 34.91141111 | -83.25384722 |
| 20_SO_11.1.1 | 143(NS) | Blount | Tennessee | Blount Co | North Smoky (NS) | 35.6711 | -83.8473 |
| 20_SO_11.1.2 | 144(NS) | Blount | Tennessee | Blount Co | North Smoky (NS) | 35.6711 | -83.8473 |
| 20_SO_11.1.3 | 145(NS) | Blount | Tennessee | Blount Co | North Smoky (NS) | 35.6711 | -83.8473 |
| 20_SO_11.1.4 | 146(NS) | Blount | Tennessee | Blount Co | North Smoky (NS) | 35.6711 | -83.8473 |
| 20_SO_11.1.5 | 147(NS) | Blount | Tennessee | Blount Co | North Smoky (NS) | 35.6711 | -83.8473 |
